# Drug-induced liver injury as a strong independent predictor of in-hospital mortality in tuberculous meningitis: potential age-related effect modification suggested in a large lifespan cohort

**DOI:** 10.3389/fmed.2026.1811720

**Published:** 2026-06-11

**Authors:** Qiong Wu, Jian Peng, Xiangzhi Xiao, Huashan Zhou, Yan Ouyang, Sufen Chen, Jue Hu, Yanhua Zhou, Tieqiao Feng, Wengao Zeng

**Affiliations:** 1 Department of Neurosurgery, The Affiliated Changsha Central Hospital, Hengyang Medical School, University of South China, Changsha, Hunan, China; 2 Department of Neurology, The Affiliated Changsha Central Hospital, Hengyang Medical School, University of South China, Changsha, Hunan, China; 3 Department of Internal Medicine, The Second Hospital of Changsha (West Branch of Changsha Hospital for Maternal & Child Health Care), Changsha, Hunan, China; 4 Department of Pathology, The Second Hospital of Changsha (West Branch of Changsha Hospital for Maternal & Child Health Care), Changsha, Hunan, China; 5 Department of Otolaryngology, The Second Hospital of Changsha (West Branch of Changsha Hospital for Maternal & Child Health Care), Changsha, Hunan, China

**Keywords:** age interaction, drug-induced liver injury, Firth logistic regression, in-hospital mortality, restricted cubic spline, tuberculous meningitis

## Abstract

**Background:**

Drug-induced liver injury (DILI) is a common complication during anti-tuberculosis treatment for tuberculous meningitis (TBM), but its association with in-hospital mortality and age-specific effect modification remains unclear.

**Methods:**

We conducted a single-center retrospective cohort of non-HIV TBM patients hospitalized between October 2013 and January 2024. DILI was ascertained using a structured retrospective adjudication workflow incorporating biochemical thresholds, temporal relationship with anti-tuberculosis therapy, exclusion of competing etiologies, and clinical evolution. The primary analysis used multivariable Firth penalized logistic regression adjusted for age, hydrocephalus, immunodeficiency, albumin, cerebrospinal fluid (CSF) glucose, and hyponatremia. Inverse probability of treatment weighting (IPTW), MRI-adjusted analysis, and sensitivity analyses were performed. Restricted cubic splines (RCS) assessed age-DILI interaction. Incremental predictive value was assessed by comparing models with and without DILI.

**Results:**

Among 1,574 patients, in-hospital mortality was 11.9% (187/1,574), and DILI occurred in 13.8% (217/1,574). DILI was independently associated with in-hospital mortality in the primary Firth model (adjusted odds ratio [aOR] 8.536, 95% confidence interval [CI] 5.970–12.245; *P* < 0.001), with consistent IPTW (weighted OR 8.699, 95% CI 6.010–12.591; *P* < 0.001) and MRI-adjusted estimates (aOR 8.617, 95% CI 6.015–12.388; *P* < 0.001). Median time from anti-tuberculosis treatment initiation to DILI onset was 23 days (IQR 18–29). Among DILI non-survivors, DILI preceded death in all cases (84/84), and no first DILI episode occurred within 48 h before death. Adding DILI increased the area under the receiver operating characteristic curve (AUC) from 0.661 to 0.805 (ΔAUC = 0.144). RCS suggested borderline overall age-DILI interaction (*P* = 0.069), without non-linear interaction (*P* = 0.835).

**Conclusion:**

DILI is a strong independent predictor of in-hospital mortality in TBM, improves mortality risk prediction, and shows an exploratory borderline trend toward age-related effect modification. Timing and sensitivity analyses suggest that this association is unlikely to be explained solely by terminal liver injury or early-death bias. These findings support intensive liver-function monitoring, prompt etiologic adjudication, and early protocolized regimen adjustment in TBM patients with DILI.

## Introduction

Tuberculous meningitis (TBM) is the most severe form of tuberculosis, with in-hospital mortality rates of 10%–30% (1–3). Anti-tuberculosis therapy frequently leads to drug-induced liver injury (DILI), a major cause of treatment interruption and poor outcomes. Previous TBM cohorts have reported drug-induced hepatitis or hepatotoxicity rates ranging from approximately 10%–42.5%, with some studies suggesting increased mortality risk among affected patients (4–6). However, whether this association varies across age groups (particularly between children and adults) remains unknown, and non-linear effects have not been systematically evaluated.

We aimed to quantify the independent association of DILI with in-hospital mortality in a large HIV-negative TBM cohort spanning the lifespan, using inverse probability of treatment weighting (IPTW) and bias-reduced estimation with Firth logistic regression to address potential separation due to the strong DILI–mortality association, and to specifically test for linear and non-linear age-related effect modification using restricted cubic splines (RCS).

## Materials and methods

### Study population

We conducted a single-center, retrospective observational cohort study of patients diagnosed with tuberculous meningitis at Changsha Central Hospital between 1 October 2013, and 1 January 2024. Eligible patients were identified from the hospital electronic medical record system according to predefined inclusion and exclusion criteria. This study was designed and reported in accordance with the Strengthening the Reporting of Observational Studies in Epidemiology (STROBE) guidelines (7). TBM was diagnosed according to internationally accepted consensus criteria, integrating clinical features, cerebrospinal fluid (CSF) characteristics, neuroimaging findings, and microbiological evidence, as previously described (8). Exclusion criteria included (1) documented HIV co-infection; (2) concomitant intracranial infections, including bacterial or fungal meningitis; and (3) excessive missingness (>30%) in key covariates required for multivariable modeling. Patients were classified into pediatric (≤18 years; *n* = 211) and adult (>18 years; *n* = 1,363) groups based on age at admission. The index date was defined as the date of first hospitalization for TBM, and follow-up was restricted to the inpatient period. The study was approved by the Ethics Committee of Changsha Central Hospital (Approval No. 20250122). The requirement for informed consent was waived by the Ethics Committee due to the retrospective nature of the study and the use of fully anonymized data, which posed no risk to patient privacy or rights. This waiver is in accordance with the ethical guidelines for biomedical research involving human subjects in China.

### Data collection and variable definitions

Demographic characteristics, clinical manifestations, laboratory findings in blood and CSF, and neuroimaging data were systematically extracted from electronic medical records by two independent investigators. Discrepancies were resolved through joint review and consensus, and data completeness and plausibility were checked before analysis.

To minimize potential reverse causation, continuous variables were recorded using values obtained at hospital admission or at the earliest time point prior to outcome occurrence.

### Outcome

The primary outcome was in-hospital mortality, defined as death from any cause during the index TBM hospitalization.

### Exposure

Drug-induced liver injury was defined as liver biochemical abnormalities occurring after hospital admission and after initiation of anti-tuberculosis therapy, temporally compatible with anti-tuberculosis treatment and not better explained by competing etiologies. Specifically, DILI was diagnosed if any of the following criteria were met during hospitalization: (1) alanine aminotransferase (ALT) ≥ 3 × the upper limit of normal (ULN) with liver-related symptoms; (2) ALT ≥ 5 × ULN irrespective of symptoms; (3) alkaline phosphatase (ALP) ≥ 2 × ULN, particularly when accompanied by elevated gamma-glutamyl transferase; or (4) total bilirubin ≥ 2 × ULN in combination with elevated ALT or ALP, after reasonable exclusion of other causes of liver injury (9).

To strengthen exposure ascertainment, DILI causality was adjudicated retrospectively using a Council for International Organizations of Medical Sciences/Roussel Uclaf Causality Assessment Method (CIOMS/RUCAM)-informed workflow rather than a formal prospective numeric RUCAM score. This workflow incorporated biochemical thresholds, temporal relationship with anti-tuberculosis treatment, exclusion of viral hepatitis and ischemic hepatitis, assessment of shock or multiorgan failure before liver injury, treatment interruption or regimen modification after DILI, hepatoprotective treatment, and subsequent liver-function evolution. Final adjudication status was recorded before sensitivity analyses. Only liver injury events occurring during the index hospitalization after anti-tuberculosis treatment initiation were included; patients with documented chronic liver disease at baseline were classified separately (10, 11).

### Covariates

Candidate covariates included demographic factors, comorbidities, laboratory parameters, and other clinically or biologically plausible predictors of TBM prognosis identified in prior literature.

Hydrocephalus was defined as ventriculomegaly on cranial computed tomography (CT) or magnetic resonance imaging (MRI), quantified by an Evans’ index greater than 0.3 when measurable, or based on radiology reports indicating TBM-related ventricular enlargement (12). Clinical relevance was assessed according to signs of raised intracranial pressure and the need for CSF diversion procedures (13). Hydrocephalus status was determined at baseline using neuroimaging performed on admission or the earliest available scan.

Altered consciousness was defined as a Glasgow Coma Scale (GCS) score < 15 at admission.

Hyponatremia was defined as a serum sodium concentration < 135 mmol/L measured within 24 h of admission (14).

Anemia was defined according to World Health Organization criteria (hemoglobin < 130 g/L in adult men, <120 g/L in adult women, and age-adjusted thresholds in children) (15).

Cerebral infarction was defined as radiologically confirmed acute or subacute ischemic lesions on CT or MRI consistent with vascular territory infarction (16, 17).

Immunodeficiency: This study focused on secondary immunodeficiency, defined as acquired immune system dysfunction associated with increased susceptibility to infections. HIV-positive patients were excluded (18, 19).

Renal failure was defined as an acute decline in kidney function, manifested by serum creatinine increase ≥ 1.5 times baseline or urine output < 0.5 mL/kg/h for 6 hours, consistent with commonly used acute kidney injury criteria (20).

Brain MRI was performed using a 1.5T or 3.0T scanner with conventional and contrast-enhanced sequences, including T1-weighted, T2-weighted, fluid-attenuated inversion recovery (FLAIR), and post-contrast imaging (21–23). Images were independently reviewed by two senior radiologists blinded to clinical and outcome information to minimize interpretation bias (23). MRI findings were categorized according to prespecified TBM-related neuroradiological patterns reported in the TBM imaging literature, including meningeal enhancement, hydrocephalus, infarction, tuberculoma, parenchymal inflammatory lesions, and spinal involvement (8, 24–26). The categories were defined as follows:

Normal: no signal abnormalities or structural alterations (22).

Parenchymal lesions: focal or diffuse abnormal parenchymal signals, including infarcts, tuberculomas, tuberculous abscess/cerebritis, or encephalitic lesions (25).

Meningeal enhancement: abnormal leptomeningeal or basal cisternal enhancement after contrast administration, reflecting inflammatory meningeal involvement (8, 25).

Complicated: multiple TBM-related complications, including hydrocephalus, infarction, edema or mass effect around tuberculomas, vasculitic changes, and, when present, hemorrhage (24, 25).

Spinal involvement: abnormal spinal cord, nerve-root, or meningeal signal/enhancement indicating spinal extension of neurotuberculosis (26).

Treatment-related variables, including anti-tuberculosis treatment exposure and regimen modification after DILI, were extracted from medical records. These treatment-related variables were interpreted in the context of contemporary tuberculosis treatment recommendations, which emphasize individualized regimen selection, monitoring of treatment-limiting drug toxicity, and modification or reintroduction of anti-tuberculosis drugs after hepatotoxicity (27–29).

### Statistical analysis

#### Descriptive analysis

Continuous variables were summarized as medians (interquartile ranges), and categorical variables as counts (percentages). Group comparisons between survivors and non-survivors were performed using the Wilcoxon rank-sum test for continuous variables and Fisher’s exact test for categorical variables, with simulated Fisher’s exact *P*-values used when appropriate. These comparisons were interpreted descriptively rather than as confirmatory hypothesis tests.

#### Model development and variable selection

Multivariable Firth penalized logistic regression using the logistf package was pre-specified as the primary model to estimate the adjusted association between DILI and in-hospital mortality while reducing small-sample bias and addressing potential complete or quasi-complete separation arising from sparse data and the strong DILI–mortality association (30). The primary model adjusted for covariates selected a priori based on previously reported prognostic factors in adult tuberculous meningitis, including age, hydrocephalus, immunodeficiency status, albumin, CSF glucose, and hyponatremia/plasma sodium (31–34). IPTW based on propensity score-derived weights was applied as a robustness analysis using the same covariates, and covariate balance was assessed by standardized mean differences and Love plots (35–37). A separate MRI-adjusted sensitivity model additionally included MRI classification. Reviewer-driven sensitivity analyses excluded patients whose first DILI episode occurred within 48 h before death, excluded early deaths within 7 days of admission, excluded DILI cases with shock or multiorgan failure before liver injury, and repeated the analysis using final-adjudicated and strict DILI definitions.

#### Assessment of the effect of DILI

Adjusted odds ratios and 95% confidence intervals for DILI were reported separately for the primary Firth model, IPTW model, MRI-adjusted sensitivity model, and reviewer-driven sensitivity analyses. Unless explicitly stated otherwise, all reported ORs for the DILI-mortality association are adjusted estimates derived from the model specified in the corresponding table or text.

#### Age effect-modification analysis

RCS with 4 knots was used to model continuous age and its interaction with DILI using the rms package, following standard recommendations for modeling non-linear associations (38). We tested the overall age-DILI interaction and the non-linear component separately by ANOVA. The interaction analysis was interpreted as exploratory because it was not the primary endpoint and because the pediatric subgroup had fewer events.

#### Model performance and internal validation

Discrimination was assessed by the apparent area under the receiver operating characteristic curve (AUC). To address the reviewer-requested AUC increment analysis, the reference model included age, hydrocephalus, immunodeficiency, albumin, CSF glucose, and hyponatremia; DILI was then added to this baseline model to quantify its incremental predictive contribution. Model performance was further evaluated by Brier score and calibration plots. Internal validation used 1,000 bootstrap resamples, repeating the modeling procedure within each resample. Supplementary prediction employed least absolute shrinkage and selection operator (LASSO) regression using cross-validated penalized logistic regression implemented in glmnet (cv.glmnet, λmin), with bootstrap validation accounting for tuning variability and model uncertainty.

For the primary Firth, IPTW, MRI-adjusted, AUC increment, and RCS analyses, complete-case datasets were used according to the variables included in each corresponding model. Missingness was assessed before modeling, and variables with excessive missingness were not included in the primary adjustment set. No imputed estimates were used for the main results reported in this manuscript. All analyses were conducted using R software (version 4.5.2). Two-sided *P*-values < 0.05 were considered statistically significant.

## Results

### Patient characteristics

**FIGURE 1:**
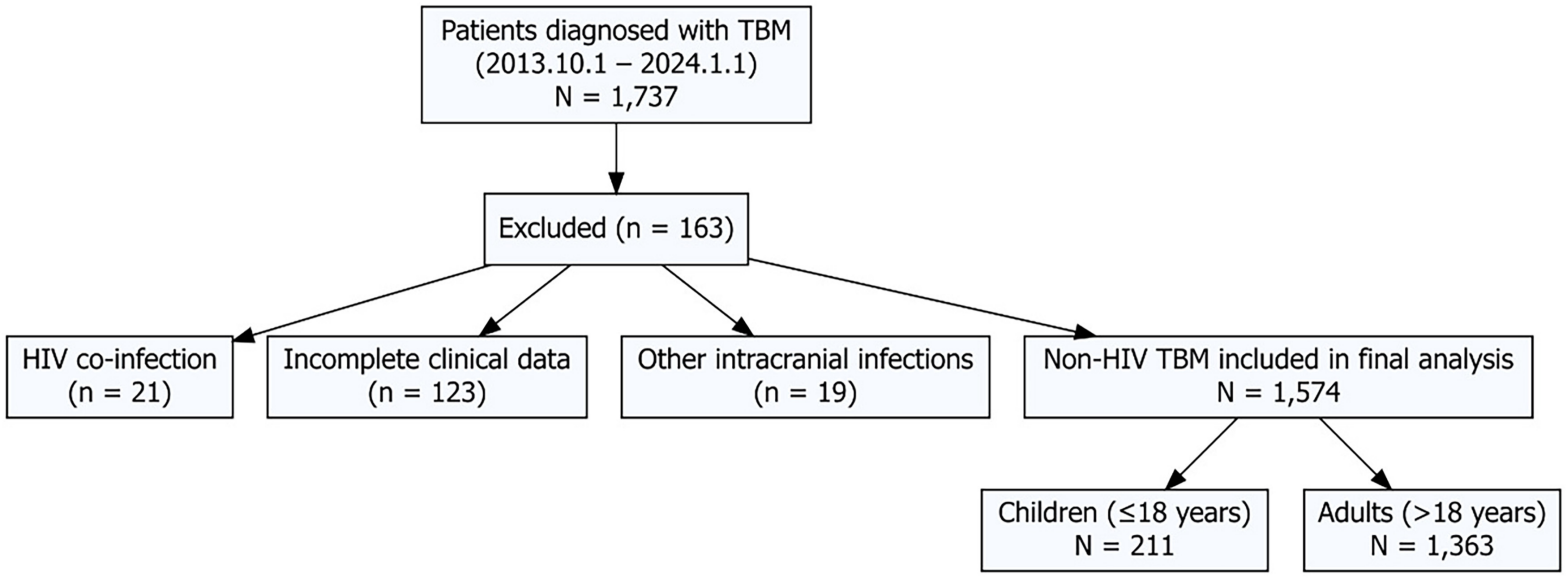
Flow diagram of patient selection. Of 1,737 patients diagnosed with tuberculous meningitis between October 2013 and January 2024, 1,574 non-HIV patients were included after excluding those with HIV co-infection (*n* = 21), incomplete data (*n* = 123), and concomitant intracranial infections (*n* = 19). The cohort comprised 211 children (≤18 years) and 1,363 adults.

A total of 1,574 non-HIV patients with TBM were included after exclusions (Figure 1). Of these, 211 (13.4%) were children (age ≤ 18 years) and 1,363 (86.6%) were adults. The overall in-hospital mortality rate was 11.9% (187/1,574), and DILI occurred in 217 patients (13.8%).

Patient characteristics stratified by in-hospital mortality are shown in Table 1. Table 1 summarizes baseline demographic, clinical, laboratory, diagnostic, and treatment characteristics stratified by in-hospital outcome, including inflammatory and coagulation variables such as C-reactive protein (CRP), D-dimer, international normalized ratio (INR), and activated partial thromboplastin time (APTT). Patients who died were more likely to have immunodeficiency (42% vs. 27%, *P* < 0.001), hyponatremia (40% vs. 26%, *P* < 0.001), renal failure (42% vs. 24%, *P* < 0.001), and DILI (45% vs. 9.6%, *P* < 0.001).

**TABLE 1:** Baseline demographic, clinical, laboratory, diagnostic, and treatment characteristics stratified by in-hospital outcome.

Variable	Overall *N* = 1,574	Survival *N* = 1,387	Death *N* = 187	*P*-value
Demographics				
Age (years), median (IQR)	44.00 (25.00, 59.00)	44.00 (25.00, 59.00)	45.00 (28.00, 62.00)	0.055
Female sex, *n* (%)	1,035 (66%)	906 (65%)	129 (69%)	0.400
Comorbidities and clinical features				
Pulmonary tuberculosis type, *n* (%)				0.600
No pulmonary tuberculosis	347 (22%)	310 (22%)	37 (20%)	–
Hematogenous dissemination	490 (31%)	422 (30%)	68 (36%)	–
Primary pulmonary tuberculosis	53 (3.4%)	48 (3.5%)	5 (2.7%)	–
Secondary pulmonary tuberculosis	679 (43%)	602 (43%)	77 (41%)	–
Tuberculous pleurisy	5 (0.3%)	5 (0.4%)	0 (0%)	–
Hepatitis B, *n* (%)	137 (8.7%)	120 (8.7%)	17 (9.1%)	0.800
DILI, *n* (%)	217 (13.8%)	133 (9.6%)	84 (45%)	<0.001
Hydrocephalus, *n* (%)	119 (7.6%)	88 (6.3%)	31 (17%)	<0.001
Cerebral hemorrhage, *n* (%)	16 (1.0%)	11 (0.8%)	5 (2.7%)	0.033
Immunodeficiency, *n* (%)	452 (29%)	374 (27%)	78 (42%)	<0.001
Cerebral infarction, *n* (%)	265 (17%)	228 (16%)	37 (20%)	0.300
Epilepsy, *n* (%)	86 (5.5%)	61 (4.4%)	25 (13%)	<0.001
Renal failure, *n* (%)	412 (26%)	334 (24%)	78 (42%)	<0.001
Hyponatremia, *n* (%)	442 (28%)	367 (26%)	75 (40%)	<0.001
Laboratory findings				
CRP (mg/L), median (IQR)	5.00 (5.00, 14.60)	5.00 (5.00, 14.10)	5.00 (5.00, 15.30)	0.010
D-dimer, median (IQR)	0.60 (0.60, 0.60)	0.60 (0.60, 0.60)	0.60 (0.60, 0.60)	0.003
INR, median (IQR)	1.00 (1.00, 1.06)	1.00 (1.00, 1.05)	1.00 (1.00, 1.10)	0.089
APTT (s), median (IQR)	28.00 (28.00, 32.20)	28.00 (28.00, 32.00)	28.00 (28.00, 34.50)	0.100
Fibrinogen (g/L), median (IQR)	4.60 (2.83, 4.60)	4.60 (2.84, 4.60)	4.60 (2.83, 4.60)	0.200
Serum uric acid (μmol/L), median (IQR)	370.50 (250.00, 524.00)	374.70 (250.00, 529.70)	296.00 (196.00, 515.00)	0.023
Serum creatinine (μmol/L), median (IQR)	48.00 (36.00, 65.00)	47.00 (36.00, 63.00)	54.40 (36.00, 78.00)	<0.001
CSF white blood cell count (×10∧6/L), median (IQR)	10.00 (10.00, 60.00)	10.00 (10.00, 51.00)	11.00 (10.00, 100.00)	0.031
Proportion of mononuclear cells in CSF, median (IQR)	0.68 (0.50, 0.69)	0.68 (0.50, 0.69)	0.67 (0.50, 0.69)	0.044
CSF chloride (mmol/L), median (IQR)	124.20 (115.00, 125.00)	125.00 (116.00, 125.00)	121.00 (113.00, 125.00)	0.016
CSF glucose (mmol/L), median (IQR)	2.20 (2.20, 3.21)	2.20 (2.20, 3.21)	2.20 (1.93, 3.21)	0.200
CSF protein (g/L), median (IQR)	0.90 (0.59, 1.37)	0.90 (0.58, 1.32)	0.91 (0.60, 1.84)	0.013
CSF ADA (U/L), median (IQR)	2.43 (2.43, 3.00)	2.43 (2.43, 2.80)	2.43 (2.43, 5.00)	0.038
Platelet count (×10∧9/L), median (IQR)	217.00 (154.00, 275.00)	217.00 (158.00, 275.00)	216.00 (125.00, 272.00)	0.052
ESR (mm/h), median (IQR)	20.00 (19.00, 39.00)	20.00 (18.00, 37.00)	20.00 (20.00, 46.00)	0.002
Hemoglobin (g/L), median (IQR)	114.15 (105.00, 126.00)	115.00 (105.00, 126.00)	110.00 (101.70, 119.00)	<0.001
PCT (ng/mL), median (IQR)	0.04 (0.03, 0.09)	0.04 (0.03, 0.08)	0.05 (0.03, 0.28)	<0.001
log(PCT), median (IQR)	−3.22 (−3.51, −2.41)	−3.22 (−3.51, −2.53)	−3.00 (−3.51, −1.27)	<0.001
Albumin (g/L), median (IQR)	35.00 (32.00, 38.50)	35.00 (32.50, 38.80)	34.30 (29.00, 37.00)	<0.001
ICP (mmH_2_O), median (IQR)	150.00 (120.00, 190.00)	150.00 (125.00, 190.00)	150.00 (120.00, 220.00)	0.200
Diagnostic tests and treatment				
Drug-resistant tuberculosis, *n* (%)	98 (6.2%)	83 (6.0%)	15 (8.0%)	0.300
PPD positivity, *n* (%)	131 (8.3%)	114 (8.2%)	17 (9.1%)	0.700
CSF tuberculosis antibody positive, *n* (%)	195 (12%)	165 (12%)	30 (16%)	0.120
CSF acid-fast bacilli smear positive, *n* (%)	7 (0.4%)	6 (0.4%)	1 (0.5%)	0.600
CSF mycobacterium tuberculosis DNA detected, *n* (%)	45 (2.9%)	40 (2.9%)	5 (2.7%)	>0.900
Aspirin use, *n* (%)	844 (54%)	737 (53%)	107 (57%)	0.300
Levofloxacin, *n* (%)	859 (55%)	758 (55%)	101 (54%)	0.900
Corticosteroid, *n* (%)	1,535 (98%)	1,351 (97%)	184 (98%)	0.600
Linezolid, *n* (%)	996 (63%)	884 (64%)	112 (60%)	0.300
Brain MRI findings, *n* (%)				0.200
Normal	226 (14%)	205 (15%)	21 (11%)	–
Parenchymal lesions	1,068 (68%)	939 (68%)	129 (69%)	–
Meningeal enhancement	160 (10%)	140 (10%)	20 (11%)	–
Complicated	81 (5.1%)	66 (4.8%)	15 (8.0%)	–
Spinal involvement	39 (2.5%)	37 (2.7%)	2 (1.1%)	–

Values are presented as median (IQR) for continuous variables and *n* (%) for categorical variables. *P*-values were calculated using the Wilcoxon rank-sum test for continuous variables and Fisher’s exact test for categorical variables; simulated Fisher’s exact *P*-values were used when appropriate. ADA, adenosine deaminase; AFB, acid-fast bacilli; ALB, albumin; APTT, activated partial thromboplastin time; CRP, C-reactive protein; CSF, cerebrospinal fluid; DILI, drug-induced liver injury; ESR, erythrocyte sedimentation rate; ICP, intracranial pressure; INR, international normalized ratio; IQR, interquartile range; PCT, procalcitonin; PPD, purified protein derivative; TBM, tuberculous meningitis.

### Association between DILI and in-hospital mortality

Firth penalized logistic regression adjusting for age, hydrocephalus, albumin, CSF glucose, immunodeficiency, and hyponatremia showed that DILI was independently associated with in-hospital mortality (aOR 8.536, 95% CI 5.970–12.245; *P* < 0.001).

**FIGURE 2:**
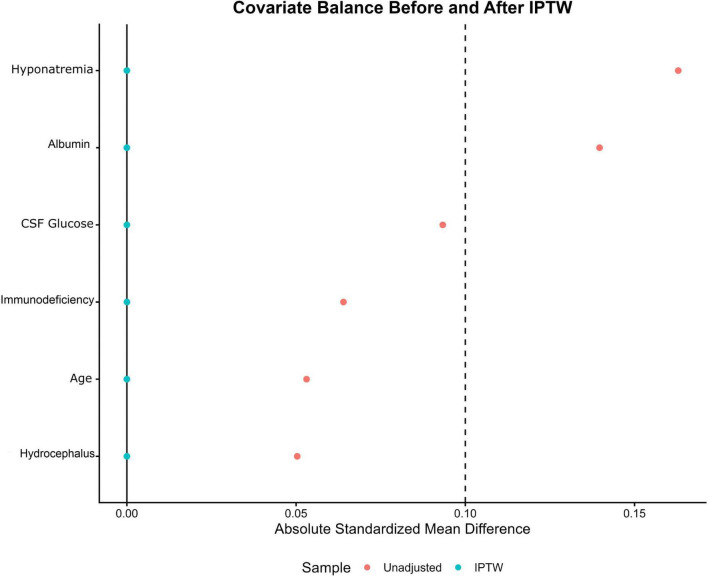
Love plot showing covariate balance before and after inverse probability of treatment weighting (IPTW). All absolute standardized mean differences were <0.10 after weighting.

To address potential confounding, IPTW was applied using the same covariates. The maximum absolute standardized mean difference was reduced from 0.140 before weighting to approximately 0 after weighting, indicating excellent covariate balance (Figure 2). The IPTW weighted analysis yielded a consistent association between DILI and mortality (weighted OR 8.699, 95% CI 6.010–12.591; *P* < 0.001). MRI-adjusted sensitivity analysis gave a similar estimate (aOR 8.617, 95% CI 6.015–12.388; *P* < 0.001; Supplementary Table 2). The E-value was 16.556 for the point estimate and 11.416 for the lower confidence limit.

Drug-induced liver injury timing, adjudication, management, and outcome evolution are summarized in Supplementary Table 1. Among DILI cases, valid interval data were available for 213 of 217 cases for onset-time calculations after the prespecified date-quality audit. The median time from admission to DILI onset was 31 days (IQR 26–40; *n* = 213), and the median time from anti-tuberculosis treatment initiation to DILI onset was 23 days (IQR 18–29; *n* = 213). The median time from DILI onset to death or discharge was 37 days (IQR 26–50; *n* = 215). Alternative causes were excluded in all DILI cases (217/217), viral hepatitis was excluded in 211/217 (97.2%), ischemic hepatitis was excluded in 217/217 (100%), and final adjudication supported DILI in 217/217 (100%). Shock or multiorgan failure before DILI was present in only 10/217 (4.6%) cases. After DILI, anti-tuberculosis treatment was interrupted in 217/217 (100%), the regimen was modified in 211/217 (97.2%), and hepatoprotective treatment was used in 217/217 (100%). Liver function improved or resolved in 135/217 (62.2%), did not improve in 38/217 (17.5%), and was not reassessed before death in 44/217 (20.3%). Among DILI non-survivors, DILI preceded death in all cases (84/84), and no first DILI episode occurred within 48 h before death.

### Predictive performance and variable selection

**FIGURE 3:**
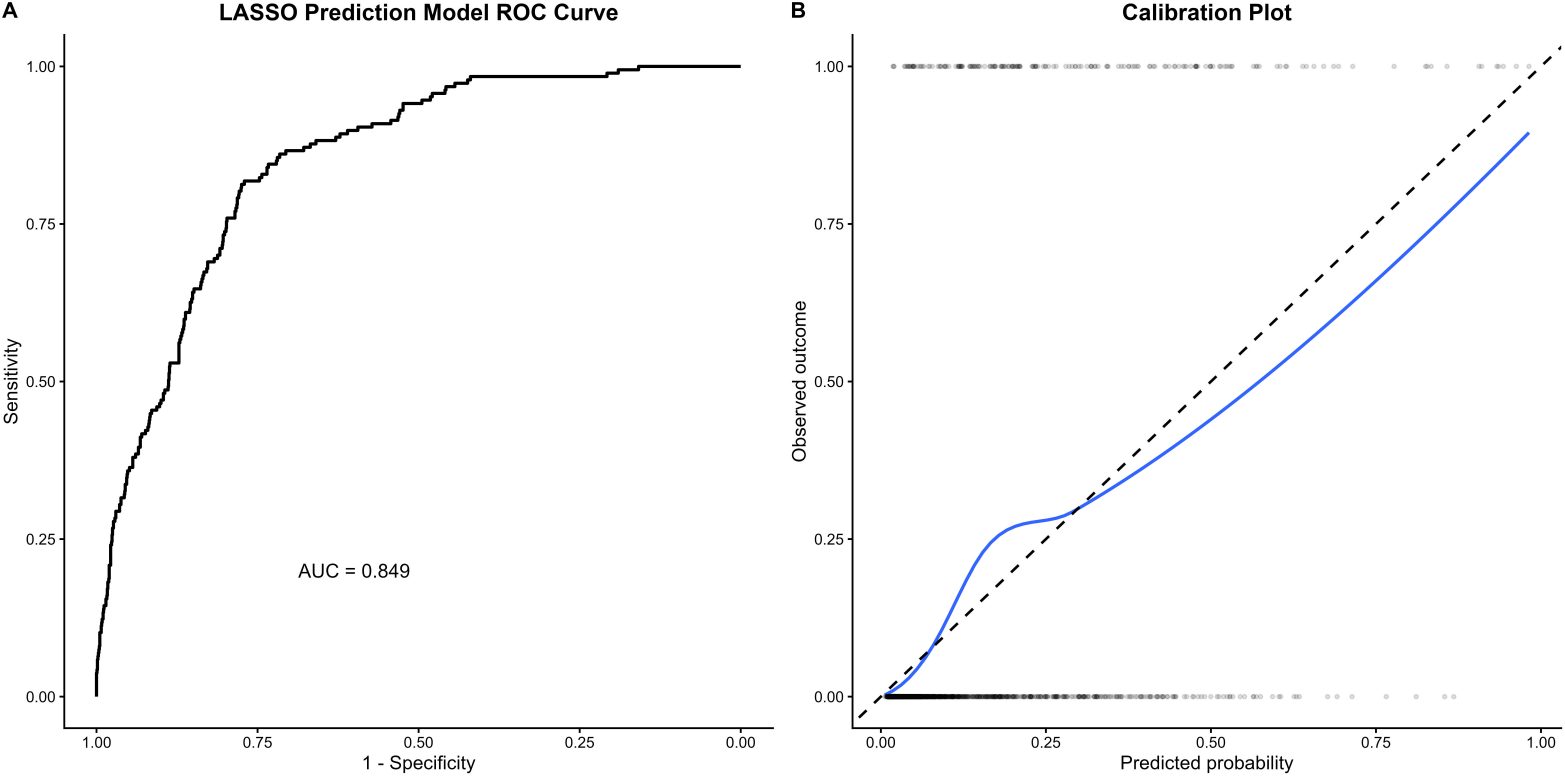
**(A)** Receiver operating characteristic (ROC) curve and **(B)** calibration plot for the LASSO-selected multivariable model predicting in-hospital mortality. Apparent AUC = 0.849; optimism-corrected AUC = 0.855; Brier score = 0.0842.

Least absolute shrinkage and selection operator regression selected 14 variables, including DILI, immunodeficiency, cerebral hemorrhage, epilepsy, hydrocephalus, hyponatremia, renal failure, uric acid, CSF protein, ESR, hemoglobin, PCT, log(PCT), and albumin. The LASSO prediction model achieved an apparent AUC of 0.849 and an optimism-corrected AUC of 0.855, with a Brier score of 0.0842 and a calibration slope of 1.00 (Figure 3). In the reviewer-requested AUC increment analysis, the baseline model included age, hydrocephalus, immunodeficiency, albumin, CSF glucose, and hyponatremia. Adding DILI increased AUC from 0.661 to 0.805 (ΔAUC = 0.144) and improved the Brier score from 0.1001 to 0.0908 (Supplementary Figure 1).

### Effect modification by age

**FIGURE 4:**
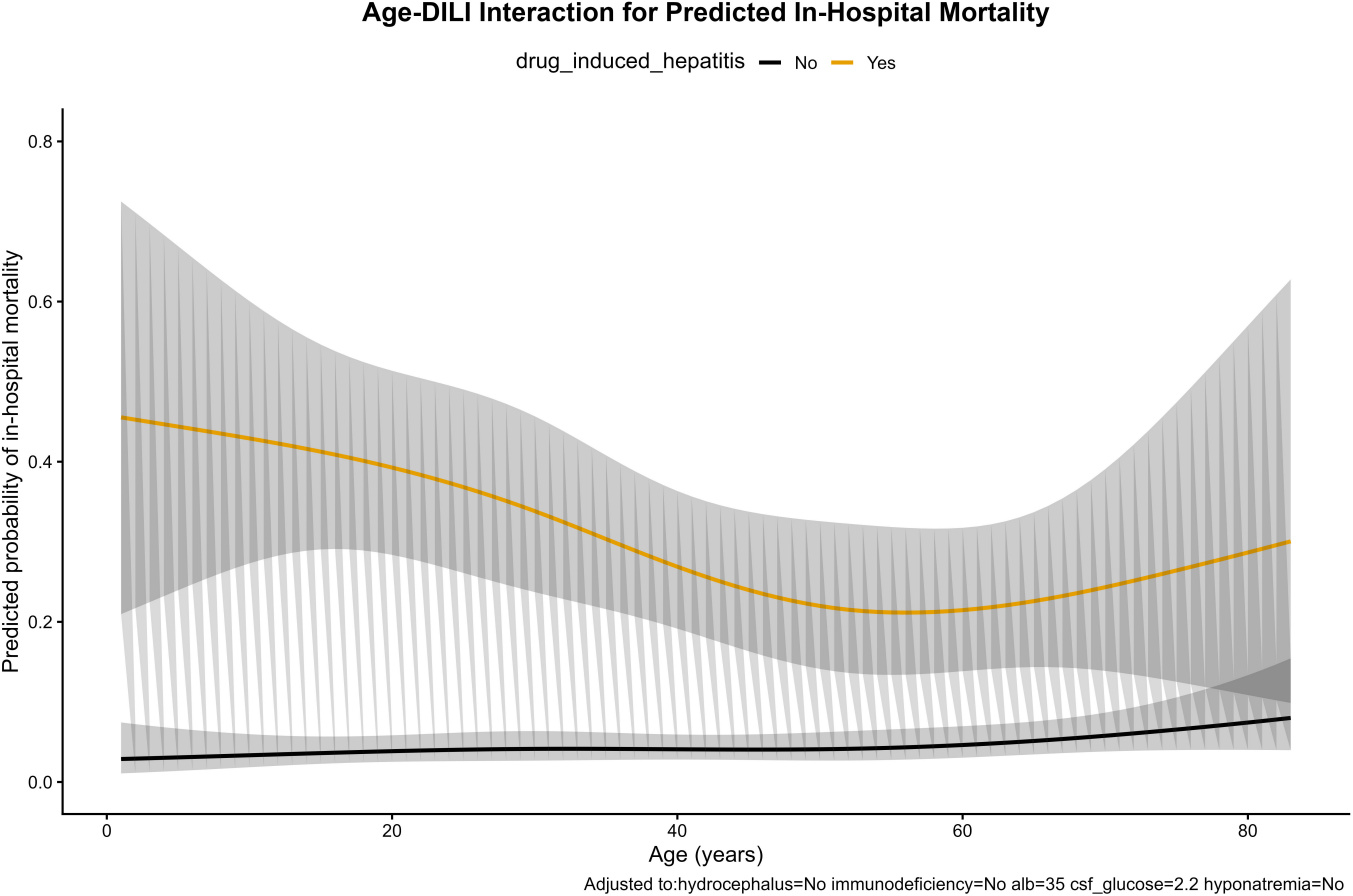
Restricted cubic spline plot of predicted mortality probability by age, stratified by drug-induced liver injury status. Shaded areas represent 95% confidence intervals. P for overall age-DILI interaction = 0.069; P for non-linear interaction = 0.835.

Restricted cubic spline analysis showed a borderline overall age-DILI interaction (*P* = 0.069). However, the non-linear component was not significant (*P* = 0.835), indicating no clear evidence of a non-linear age-dependent DILI effect. Predicted mortality curves suggested a directionally steeper mortality increase with age among patients with DILI, but this exploratory finding should be interpreted cautiously (Figure 4).

## Discussion

In this large retrospective lifespan cohort, DILI was independently associated with in-hospital mortality, with an aOR of 8.536 in the primary Firth-penalized model and a weighted OR of 8.699 in the IPTW analysis. The association remained consistent across MRI-adjusted and reviewer-driven sensitivity analyses. The revised timing analysis showed that DILI typically developed after a clinically meaningful interval following treatment initiation (median 23 days after anti-tuberculosis therapy), preceded death among all DILI non-survivors, and was not merely a terminal event occurring within 48 h before death.

Reports on DILI in TBM have been heterogeneous, with pediatric evidence remaining limited compared with adult data.

In adult or predominantly adult TBM studies from high-burden settings, DILI has been associated with higher mortality, treatment disruption, or composite poor outcomes (5, 6, 39). This association may be partly explained by treatment interruption or modification, as well as stress-related biological pathways, including oxidative stress and endoplasmic reticulum stress, which have been implicated in DIH severity, poor outcome, and mortality in TBM (40–42). Incidence rates of DILI among patients with TBM have been reported to be substantial in adult cohorts from high-burden settings. In a TBM-specific cohort of 67 patients, drug-induced hepatitis occurred in 32.8% of cases, and mortality was significantly higher among patients with DILI compared with those without DILI (32% vs. 7%; *P* = 0.01) (5). Hepatitis B co-infection has been associated with increased DILI risk and poor outcomes in TBM (39). Meta-analytic data from broader tuberculosis populations indicate that anti-tuberculosis DILI risk is higher in patients with HBV or HCV co-infection and may approach 39% in selected high-risk subgroups (43–45).

Oxidative stress and endoplasmic reticulum stress markers have been implicated in the severity of drug-induced hepatitis in adult or predominantly adult cohorts with tuberculous meningitis, supporting a biologically plausible link between hepatotoxicity and systemic stress responses in severe central nervous system tuberculosis (5, 46). In addition, hepatitis B virus infection is an established risk factor for anti-tuberculosis DILI and may further contribute to DILI-related adverse outcomes (39, 44, 47). Trials of intensified rifampicin-containing anti-tuberculosis regimens have yielded mixed safety signals. In a large phase 3 trial of high-dose rifampin for adult TBM, DILI occurred more frequently in the high-dose arm than in the standard-dose arm, but no deaths were attributed to DILI. Earlier pooled analyses did not show statistically significant differences in severe liver toxicity or hepatotoxicity between high-dose and standard-dose rifampicin regimens, although more recent meta-analytic data suggest that very high-dose regimens may increase hepatic severe adverse events. Taken together, these findings suggest that DILI may be clinically manageable in selected trial settings with close biochemical monitoring and timely treatment interruption or modification, but should not be considered benign (48–52).

Evidence in pediatric TBM remains limited. Available pediatric TBM series and pharmacokinetic studies suggest that DILI is less well-characterized in children than in adults and may be less frequent overall; when DILI occurs in pediatric TBM, limited PK data suggest an association with higher plasma exposures and temporary interruption or modification of anti-tuberculosis therapy (53–56). In a prospective pharmacokinetic study of children and adolescents with TBM, grade 2–3 DILI was observed in a subset of patients. In these patients, day-10 plasma AUC0–24 values for isoniazid, rifampicin, and pyrazinamide, together with C_*max*_ values for isoniazid and pyrazinamide, were higher than in patients without DILI, suggesting a possible exposure–toxicity relationship in pediatric TBM that requires confirmatory studies (55). Consistent with World Health Organization guidance and pediatric anti-tuberculosis hepatotoxicity reviews, adverse events caused by anti-tuberculosis medicines appear less frequent in children and adolescents than in adults, although hepatotoxicity remains clinically important and may be caused by isoniazid, rifampicin, or pyrazinamide (56, 57). However, available pediatric TBM studies have not demonstrated a consistent independent association between DILI and mortality. This should be interpreted cautiously, because existing pediatric data are limited by small sample sizes, low DILI or mortality event counts, and incomplete adjustment for hepatotoxicity rather than proving absence of effect (54, 55, 58–60).

Our study extends the existing literature by demonstrating a substantially stronger and independent association between DILI and in-hospital mortality in a large lifespan cohort. Although the overall age-DILI interaction was borderline (*P* = 0.069), the non-linear component was not significant (*P* = 0.835). Therefore, age-related effect modification should be regarded as hypothesis-generating rather than definitive. Clinically, the pattern suggests that older patients with DILI may warrant particularly close surveillance, but intensive liver-function monitoring remains relevant across the full age spectrum.

From a clinical perspective, these findings underscore the importance of proactive liver-function monitoring across all patients with TBM, coupled with early, protocolized regimen adjustment aimed at minimizing prolonged interruption of core anti-tuberculosis agents. In our cohort, most DILI cases underwent anti-tuberculosis regimen modification (97.2%) and all received treatment interruption and hepatoprotective treatment, reflecting the substantial clinical disruption caused by DILI during TBM management. The large improvement in discrimination after adding DILI to the baseline model (ΔAUC = 0.144) supports its value for dynamic in-hospital risk stratification.

Key strengths of this study include the large cohort spanning the full age spectrum, explicit DILI adjudication, strengthened timing analysis, penalized estimation to address separation, IPTW for confounding robustness, sensitivity analyses targeting reverse causation and competing causes of liver injury, internal validation, calibration assessment, and systematic evaluation of age-DILI interaction.

Limitations include the single-center retrospective design, which may introduce selection bias and limit generalizability. Although DILI timing was reconstructed from medical records and date parsing was audited, exposure ascertainment remained retrospective, and DILI was not modeled as a fully time-varying covariate. Formal prospective numeric RUCAM scoring was not available; therefore, we used a CIOMS/RUCAM-informed retrospective adjudication workflow rather than claiming prospective RUCAM assessment. Residual and unmeasured confounding cannot be excluded despite IPTW, sensitivity analyses, and a high E-value. Follow-up was restricted to the index hospitalization, so post-discharge death, delayed recovery, relapse, or long-term neurological outcomes could not be evaluated. Finally, the pediatric subgroup and interaction analyses had limited precision, and the borderline age-DILI interaction should be validated in multicenter prospective cohorts.

Prospective multicenter studies are warranted to validate these findings and to determine whether targeted prevention and early management of DILI can translate into improved survival in TBM.

## Conclusion

Drug-induced liver injury is a strong independent predictor of in-hospital mortality in TBM, with directionally consistent effects across the lifespan and a borderline trend toward age-related effect modification (P for interaction = 0.069) that remains exploratory. These findings support intensive liver-function monitoring and prompt regimen adjustment in all TBM patients, including both pediatric and adult patients, to enable early intervention and potentially improve survival outcomes.

## Data Availability

The original contributions presented in this study are included in the article/Supplementary material, further inquiries can be directed to the corresponding authors.

## Supplementary material

Supplementary Figure 1 Comparison of receiver operating characteristic (ROC) curves for prediction models with and without drug-induced liver injury. The baseline model included age, hydrocephalus, immunodeficiency, albumin, CSF glucose, and hyponatremia. AUC without DILI = 0.661; AUC with DILI = 0.805; ΔAUC = 0.144.
