# Supplementary material for: Drug-induced liver injury as a strong independent predictor of in-hospital mortality in tuberculous meningitis: potential age-related effect modification suggested in a large lifespan cohort
Source: Front Med (Lausanne). 2026 Jun 11;13:1811720. doi: 10.3389/fmed.2026.1811720 (PMC13307109; doi:10.3389/fmed.2026.1811720)
Supplement: Supplementary file 1 [file Table_1.docx]

**Supplementary Table 1. DILI timing, adjudication, management, and outcome evolution**

| **Domain** | **Variable** | **Result** |
| --- | --- | --- |
| Timing | Time from admission to DILI onset | n = 213; median 31 days (IQR 26-40; range 7-83) |
| Timing | Time from anti-TB treatment initiation to DILI onset | n = 213; median 23 days (IQR 18-29; range 0-55) |
| Timing | Time from DILI onset to death/discharge | n = 215; median 37 days (IQR 26-50; range 0-80) |
| Adjudication | Alternative causes excluded | 217/217 (100%) |
| Adjudication | Viral hepatitis excluded | 211/217 (97.2%) |
| Adjudication | Ischemic hepatitis excluded | 217/217 (100%) |
| Adjudication | Shock before DILI | 5/217 (2.3%) |
| Adjudication | Multiorgan failure before DILI | 5/216 (2.3%) |
| Adjudication | Final adjudication as DILI | 217/217 (100%) |
| Causality category | Probable / highly probable / possible | 111/217 (51.2%) / 78/217 (35.9%) / 28/217 (12.9%) |
| Management | Anti-TB regimen modified after DILI | 211/217 (97.2%) |
| Management | Anti-TB treatment interrupted after DILI | 217/217 (100%) |
| Management | Hepatoprotective treatment used | 217/217 (100%) |
| Outcome relationship | DILI preceded death among DILI non-survivors | 84/84 (100%) |
| Outcome relationship | First DILI within 48 h before death among DILI non-survivors | 0/84 (0%) |
| Evolution | DILI improved/resolved | 135/217 (62.2%) |
| Evolution | DILI not improved before death/discharge | 38/217 (17.5%) |
|  | Not reassessed before death | 44/217 (20.3%) |

Note: data are presented as n/N (%) or median (interquartile range [IQR]). Valid interval data were available for 213 of 217 adjudicated DILI cases for onset-time calculations and for 215 of 217 cases for DILI-to-outcome interval calculations after the prespecified date-quality audit. All percentages are calculated using the available data for each variable. DILI = drug-induced liver injury; Anti-TB = anti-tuberculosis; MOF = multiorgan failure.

**Supplementary Table 2. Model definitions and sensitivity analyses for DILI-mortality association**

| **Analysis** | **n** | **Events** | **Adjusted OR (95% CI)** | **P value** |
| --- | --- | --- | --- | --- |
| Primary Firth model | 1,573 | 187 | 8.536 (5.970-12.245) | <0.001 |
| IPTW weighted model | 1,573 | 187 | 8.699 (6.010-12.591) | <0.001 |
| MRI-adjusted sensitivity model | 1,573 | 187 | 8.617 (6.015-12.388) | <0.001 |
| Excluding first DILI within 48 h before death | 1,569 | 187 | 8.808 (6.150-12.660) | <0.001 |
| Excluding deaths within 7 days of admission | 1,573 | 187 | 8.536 (5.970-12.245) | <0.001 |
| Excluding DILI cases with shock or MOF before DILI | 1,563 | 184 | 8.692 (6.050-12.530) | <0.001 |
| Exposure defined by final adjudication | 1,573 | 187 | 8.536 (5.970-12.245) | <0.001 |
| Strict DILI definition | 1,573 | 187 | 8.536 (5.970-12.245) | <0.001 |

Note: the primary and sensitivity models adjusted for age, hydrocephalus, immunodeficiency, albumin, CSF glucose, and hyponatremia unless otherwise stated. IPTW used the same covariates for treatment-weight estimation. MOF, multiorgan failure. No patients meeting the prespecified early-death exclusion criterion were removed; therefore, this sensitivity analysis yielded estimates identical to the primary model. The strict DILI definition identified the same exposure set as the primary DILI definition in this dataset; therefore, the estimates were identical.
