# Supplementary figures and images for: Drug-induced liver injury as a strong independent predictor of in-hospital mortality in tuberculous meningitis: potential age-related effect modification suggested in a large lifespan cohort
Source: Front Med (Lausanne). 2026 Jun 11;13:1811720. doi: 10.3389/fmed.2026.1811720 (PMC13307109; doi:10.3389/fmed.2026.1811720)

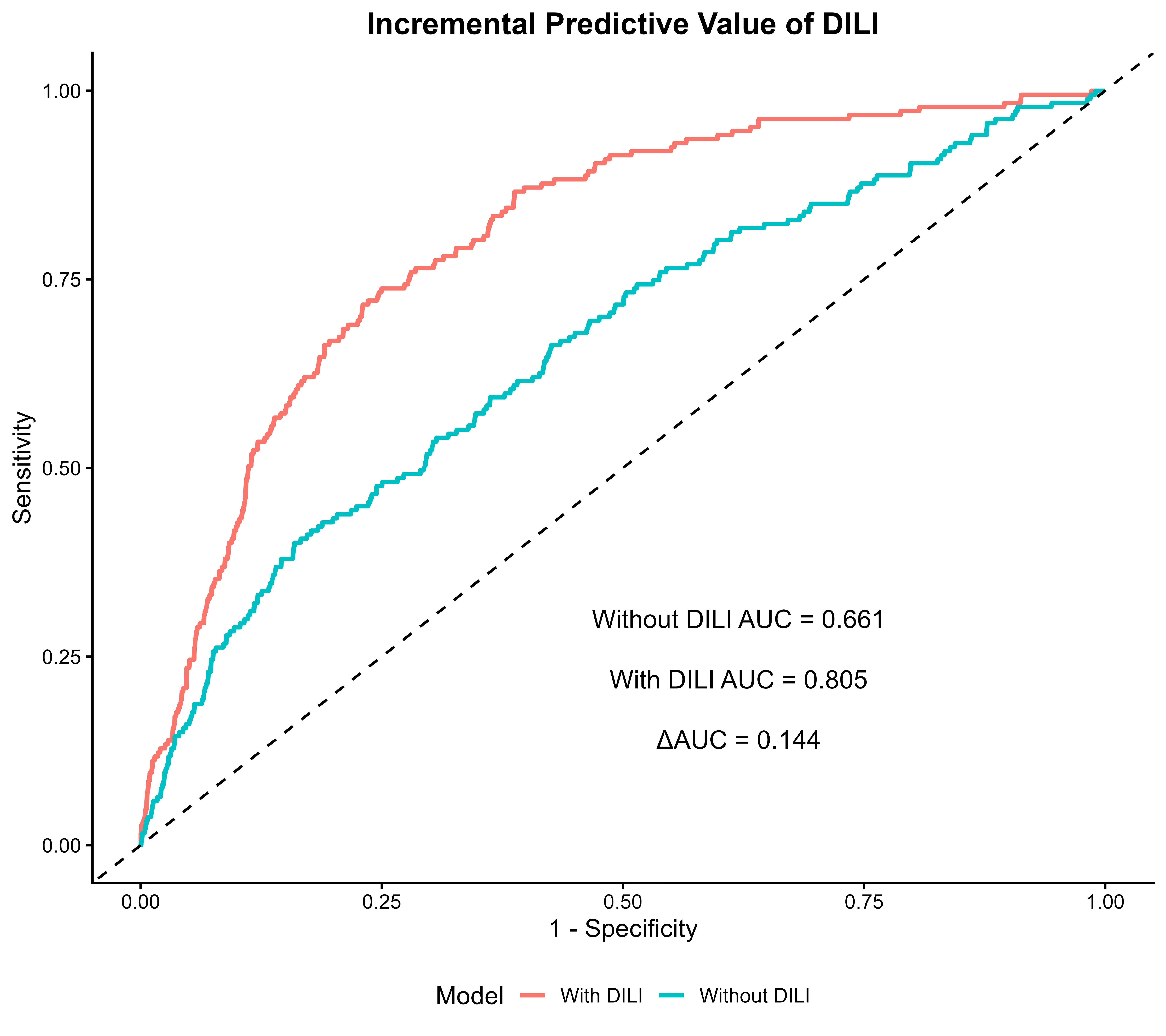

Supplement: Supplementary Figure 1 — Comparison of receiver operating characteristic (ROC) curves for prediction models with and without drug-induced liver injury. The baseline model included age, hydrocephalus, immunodeficiency, albumin, CSF glucose, and hyponatremia. AUC without DILI = 0.661; AUC with DILI = 0.805; ΔAUC = 0.144. [file Image_1.jpeg]
